# Factors in Deciding between Novel and Traditional Oral Anticoagulants to Prevent Embolism in Atrial Fibrillation Patients

**DOI:** 10.5935/abc.20160010

**Published:** 2016-01

**Authors:** Maurício Scanavacca, Francisco Darrieux

**Affiliations:** Unidade de Arritmias Cardíacas - InCor - HC - FMUSP, São Paulo, SP - Brazil

**Keywords:** Anticoagulants, Prevention, Atrial Fibrillation, Embolism and Thrombosis, Stroke.

Atrial fibrillation (AF) is the sustained arrhythmia most frequently found in clinical practice. Its prevalence is expected to increase in the coming decades. Its occurrence implies a reduction in the quality of life and an increase in mortality, mainly due to stroke and systemic thromboembolism (TE). The stroke originating from AF carries a higher risk of severe complications, such as permanent disability and prolonged hospitalization, as compared to that of other etiologies.^1-3^

Since the discovery of vitamin K (VitK) antagonists more than 50 years ago, they have become the most effective treatment to prevent stroke and TE in patients with AF. However, because of the risk of hemorrhagic complications they pose, only patients with persistent AF considered of very high risk, previous embolic accidents, mechanical valvular prostheses, and those undergoing electrical cardioversion used to receive that treatment in an initial phase. Between the 1980 and 1990 decades, major clinical controlled studies determining the importance of stroke prevention in non-valvular AF were carried out, providing scientific support to the current clinical use of VitK antagonists. The greater benefit of the VitK antagonists as compared to placebo (a mean 64% reduction in relative risk) has been undoubtedly demonstrated, as has been the modest or even absent role of acetylsalicylic acid in stroke prevention in that population.^4^

Despite that evidence, the clinical use of VitK antagonists remained very limited over the following years, because of their complex pharmacokinetics and pharmacodynamics. Undesirable drug interactions and their narrow therapeutic window (borderline between efficacy in embolism prevention and risk of bleeding) are the major limitations of their use and the reason for the need to monitor often the anticoagulation level.^5-7^

On the other hand, the advances in knowing the risk factors for the formation of AF-related atrial thrombus and embolism and the risk of bleeding due to VitK antagonists have motivated the development of new strategies based on the risk-benefit ratio of using anticoagulants to prevent stroke.^8-11^ The major risk scores currently used are CHA_2_DS_2_VASc for embolism, and HASBLED for bleeding. The balance between those two scores has made the use of anticoagulants easier. Nevertheless, VitK antagonists have been underused in clinical practice. Real world studies have shown that only 50% of the patients with indication for their use received medical recommendation, and only 50% of them (specially in Brazil) had proper INR control.^12-14^

Aiming at a better safety profile, with fewer drug and food interactions, non-VitK antagonist oral anticoagulants, the "novel oral anticoagulants" (NOACs), have been developed. Dabigatran, a direct thrombin inhibitor, was the first NOAC registered and approved by the major drug regulatory agencies around the world, based on the results of the RE-LY study in 2009.^15^ Subsequently, NOACs belonging to the family of activated factor X inhibitors were developed, being approved for clinical use by the major drug regulatory agencies around the world after the publication of the following studies: ROCKET-AF (rivaroxaban);^16^ ARISTOTLE (apixaban);^17^ and, more recently, ENGAGE (edoxaban).^18^

Considering the high efficacy of warfarin as compared to placebo and acetylsalicylic acid to prevent TE phenomena, those four studies were designed for the non-inferiority hypothesis. The results obtained with a large number of patients (70,000) have shown that NOACs are at least non-inferior to warfarin regarding efficacy. On the other hand, an unequivocal comparison could not be established between the different NOACs, because the studies are not identical. On indirect analysis, dabigatran at the dose of 150 mg, twice a day, and apixaban at the dose of 5 mg, twice a day, stood out, showing superiority over warfarin in reducing total stroke. Regarding safety, all NOACs were superior to warfarin in reducing hemorrhagic stroke and potentially fatal hemorrhages. Rivaroxaban and edoxaban stood out because of their convenient administration, with just one daily intake. Based on those clinical studies, the European Guideline of Cardiology recommends any NOAC (dabigatran, apixaban, rivaroxaban) as an alternative to VitK antagonists in patients with non-valvular AF^19^ The American guideline for the management of patients with AF recommends VitK antagonists as class IA and the NOACs (apixaban, dabigatran and rivaroxaban) as class IB for patients with non-valvular AF and risk factors for stroke and systemic embolism.^20^

Real world observations have reproduced the initial clinical studies, confirming that NOACs are an effective alternative for stroke/TE prevention in patients with AF,^21^ being also recommended by the Brazilian Society of Cardiology guideline on anticoagulation.^22^ This wider range of choice, however, generates natural questioning about the current role of VitK antagonists. Two aspects have guided the selection of anticoagulants in this transition phase, in which clinicians acquire experience with the new drugs, comparing them with those traditionally used: 1) technical questions, related to drug efficacy and safety; and 2) the possibility that the patients pay for their treatment or have it paid for by health care services.

Regarding the technical question, the advantages of warfarin are as follows: 1) it is the one and only drug with proven efficacy in patients with mitral stenosis, patients with metal valve prostheses and renal failure; 2) greater experience over decades (50 years of use); 3) the physician follows the effectiveness or risk of the treatment by controlling INR; 4) easily maintained treatment because of the low cost of the medication; 5) possibility of effect attenuation by administrating vitK or blood derivative products; and 6) prolonged therapeutic effect, so that skipping one dose usually does not interfere with the therapeutic activity. Regarding the technical question, the advantages of the NOACs are as follows: 1) rapid onset and end of their anticoagulant effect; 2) they usually do not require transition with low-molecular-weight heparin; 3) low drug interaction; 4) no food interaction; 5) important reduction in the risk for hemorrhagic stroke; and 6) smaller necessity for periodical laboratory control (although anticoagulation control is not recommended, regular renal function monitoring is still required).

There are some gray areas in the use and indication of NOACs, such as the procedures of cardioversion and ablation and the context of acute coronary disease, and the interventions with bare-metal and drug-eluting stent implantation. Regarding the cardioversions for AF, the substudies RE-LY, ARISTOTLE and ROCKET-AF have shown similar effectiveness between NOACs and VitK inhibitors, an observation confirmed by the X-VeRT trial, which randomized rivaroxaban and VitK antagonists in patients with AF undergoing cardioversion.^23^ Regarding AF ablation, isolated studies have shown that NOACs are usually effective and safe, depending on the type of protocol used. There are new ongoing studies to define the best strategy for patients in that condition.

Regarding patients with AF in the context of acute coronary disease, recently or during hemodynamic interventions, prospective studies on NOACs are awaited. The subanalyses of previous multicenter studies have not authorized the unrestricted use of NOACs in those patients, warfarin being the most often studied drug, in double or triple combination with antiplatelet agents. However, there are "recommendations" for the early use of NOACs at their lowest dose studied (rivaroxaban 15 mg, once a day; dabigatran 110 mg, twice a day; or apixaban 2.5 mg, twice a day) in association with an antiplatelet agent, preferably clopidogrel.^19^ The future results of the studies conducted with that purpose will or will not support the current orientation.

A very important aspect for the incorporation of NOACs is their cost, much higher than that of VitK antagonists. This has relevant clinical implications, because their suspension, even if transient, places the patient at risk for embolic events due to the rapid loss of their anticoagulant effects and the possibility of paradoxical hypercoagulability. In the social context, most patients treated at public hospitals receive VitK antagonists. The incorporation of new anticoagulants should promote a significant impact on the budget of those hospitals.^24,25^ Therefore, Brazilian studies assessing the local needs and the clinical and financial impact of the introduction of those new therapeutic strategies in patients with AF are required. In addition, it is worth noting that, while that cost/effectiveness ratio has not been clarified, the prevention of embolism in AF by using VitK antagonists is well established, and the maintenance of INR within the therapeutic range promotes efficacy levels equivalent to those of NOACs.

In conclusion, the knowledge acquired with the management of VitK antagonists over the years allows us to glimpse a horizon of opportunities to use NOACs to perfect the prevention of TE phenomena in patients with AF. The comfort provided by NOACs, by not requiring anticoagulation level monitoring, however, should not be interpreted as no need for drug surveillance and for periodical care of the patient as a whole. Further clinical studies conducted in Brazil are required to allow the identification of patients' profiles more favorable to each of those new drugs, considering a good cost/effectiveness ratio.

## References

[1] Go AS, Hylek EM, Phillips KA, Chang Y, Henault LE, Selby JV (2001). Prevalence of diagnosed atrial fibrillation in adults: national
implications for rhythm management and stroke prevention: the
AnTicoagulation and Risk Factors in Atrial Fibrillation (ATRIA)
Study. JAMA.

[2] Almeida ED, Guimarães RB, Stephan LS, Medeiros AK, Foltz K, Santanna RT (2015). Clinical differences between subtypes of atrial fibrillation and
flutter: cross-sectional registry of 407 patients. Arq Bras Cardiol.

[3] Ferreira C, Providência R, Ferreira MJ, Gonçalves LM (2015). Atrial fibrillation and non-cardiovascular diseases: a systematic
review. Arq Bras Cardiol.

[4] Hart RG, Pearce LA, Aguilar MI (2007). Meta-analysis: antithrombotic therapy to prevent stroke in
patients who have nonvalvular atrial fibrillation. Ann Intern Med.

[5] Esmerio FG, Souza EN, Leiria TL, Lunelli R, Moraes MA (2009). Constant use of oral anticoagulants: implications in the control
of their adequate levels. Arq Bras Cardiol.

[6] Oliveira LH, Mallmann FB, Botelho FN, Paul LC, Gianotto M, Abt Rde B (2012). Cross-sectional study of treatment strategies on atrial
fibrillation. Arq Bras Cardiol.

[7] van der Sand CR, Leiria TL, Kalil RA (2013). Assessment of the adherence of cardiologists to guidelines for
the treatment of atrial fibrillation. Arq Bras Cardiol.

[8] Lip GY, Nieuwlaat R, Pisters R, Lane D, Crijns H (2010). Refining clinical risk stratification for predicting stroke and
thromboembolism in atrial fibrillation using a novel risk factor based
approach: the Euro Heart Survey on Atrial Fibrillation. Chest.

[9] Lip GY, Frison L, Halperin JL, Lane DA (2011). Comparative validation of a novel risk score for predicting
bleeding risk in anticoagulated patients with atrial fibrillation: the
HAS-BLED (Hypertension, Abnormal Renal/Liver Function, Stroke, Bleeding
History or Predisposition, Labile INR, Elderly, Drugs/Alcohol Concomitantly)
score. J Am Coll Cardiol.

[10] Santos C, Pereira T, Conde J (2013). CHADS2 score in predicting cerebrovascular events: a
meta-analysis. Arq Bras Cardiol.

[11] Lavitola P de L, Spina GS, Sampaio RO, Tarasoutchi F, Grinberg M (2009). Bleeding during oral anticoagulant therapy: warning against a
greater hazard. Arq Bras Cardiol.

[12] Fornari LS, Calderaro D, Nassar IB, Lauretti C, Nakamura L, Bagnatori R (2007). Misuse of antithrombotic therapy in atrial fibrillation patients:
frequent, pervasive and persistent. J Thromb Thrombolysis.

[13] Gamra H, Murin J, Chiang CE, Naditch-Brûlé L, Brette S, Steg PG, Realise AF investigators (2014). Use of antithrombotics in atrial fibrillation in Africa, Europe,
Asia and South America: insights from the international Realise AF
survey. Arch Cardiovasc Dis.

[14] Mendes Fde S, Atié J, Garcia MI, Gripp Ede A, Sousa AS, Feijo LA (2014). Atrial fibrillation in decompensated heart failure: associated
factors and in-hospital outcome. Arq Bras Cardiol.

[15] Connolly SJ, Ezekowitz MD, Yusuf S, Eikelboom J, Oldgren J, Parekh A, RE-LY Steering Committee and Investigators (2009). Dabigatran versus warfarin in patients with atrial
fibrillation. N Engl J Med.

[16] Patel MR, Mahaffey KW, Garg J, Pan G, Singer DE, Hacke W, ROCKET AF Investigators (2011). Rivaroxaban versus warfarin in nonvalvular atrial
fibrillation. N Engl J Med.

[17] Connolly SJ, Eikelboom J, Joyner C, Diener HC, Hart R, Golitsyn S, AVERROES Steering Committee and Investigators (2011). Apixaban in patients with atrial fibrillation. N Engl J Med.

[18] Ruff CT, Giugliano RP, Antman EM, Crugnale SE, Bocanegra T, Mercuri M (2010). Evaluation of the novel factor Xa inhibitor edoxaban compared
with warfarin in patients with atrial fibrillation: design and rationale for
the Effective aNticoaGulation with factor xA next GEneration in Atrial
Fibrillation-Thrombolysis In Myocardial Infarction study 48 (ENGAGE AF-TIMI
48). Am Heart J.

[19] Heidbuchel H, Verhamme P, Alings M, Antz M, Hacke W, Oldgren J (2013). EHRA practical guide on the use of new oral anticoagulants in
patients with non-valvular atrial fibrillation: executive
summary. Eur Heart J.

[20] January CT, Wann LS, Alpert JS, Calkins H, Cigarroa JE, Cleveland JC, ACC/AHA Task Force Members (2014). 2014 AHA/ACC/HRS guideline for the management of patients with
atrial fibrillation: executive summary: a report of the American College of
Cardiology/American Heart Association Task Force on practice guidelines and
the Heart Rhythm Society. Circulation.

[21] Larsen TB, Potpara T, Dagres N, Proclemer A, Sciarrafia E, Blomström-Lundqvist C, Scientific Initiative Committee, European Heart Rhythm Association (2015). Preference for oral anticoagulation therapy for patients with
atrial fibrillation in Europe in different clinical situations: results of
the European Heart Rhythm Association Survey. Europace.

[22] Lorga AM, Azmus AD, Soeiro AM, Quadros AS, Avezum A, Marques AC, Sociedade Brasileira de Cardiologia (2013). Brazilian guidelines on platelet antiaggregants and
anticoagulants in cardiology. Arq Bras Cardiol.

[23] Cappato R, Ezekowitz MD, Klein AL, Camm AJ, Ma CS, Le Heuzey JY, X-VeRT Investigators (2014). Rivaroxaban vs. vitamin K antagonists for cardioversion in atrial
fibrillation. Eur Heart J.

[24] Kountz DS, Shaya FT, Gradman AH, Puckrein GA, Kim MH, Wilbanks J (2015). A call for appropriate evidence and outcomes-based use and
measurement of anticoagulation for atrial fibrillation: moving the
population towards improved health via multiple stakeholders. J Manag Care Spec Pharm.

[25] Janzic A, Kos M (2015). Cost effectiveness of novel oral anticoagulants for stroke
prevention in atrial fibrillation depending on the quality of warfarin
anticoagulation control. Pharmacoeconomics.

